# Application of a Novel Collection of Exhaled Breath Condensate to Exercise Settings

**DOI:** 10.3390/ijerph19073948

**Published:** 2022-03-26

**Authors:** Joseph A. Sol, John C. Quindry

**Affiliations:** School of Integrative Physiology and Athletic Training, University of Montana, Missoula, MT 59812, USA; joseph.sol@umontana.edu

**Keywords:** biosamples, exercise, exhaled breath condensate

## Abstract

The collection of exhaled breath condensate (EBC) is a non-invasive method for obtaining biosamples from the lower respiratory tract, an approach amenable to exercise, environmental, and work physiology applications. The purpose of this study was to develop a cost-effective, reproducible methodology for obtaining larger volume EBC samples. Participants (male: *n* = 10; female: *n* = 6; 26 ± 8 yrs.) completed a 10 min EBC collection using a novel device (N-EBC). After initial collection, a 45 min bout of cycling at 75% HRmax was performed, followed by another N-EBC collection. In a subset of individuals (*n* = 5), EBC was obtained using both the novel technique and a commercially available EBC collection device (R-EBC) in a randomized fashion. N-EBC volume—pre- and post-exercise (2.3 ± 0.8 and 2.6 ± 0.9 mL, respectively)—and pH (7.4 ± 0.5 and 7.4 ± 0.5, respectively) were not significantly different. When normalized for participant body height, device comparisons indicated N-EBC volumes were larger than R-EBC at pre-exercise (+12%) and post-exercise (+48%). Following moderate-intensity exercise, no changes in the pre- and post-trial values of Pentraxin 3 (0.25 ± 0.04 and 0.26 ± 0.06 pg/mL, respectively) and 8-Isoprostrane (0.43 ± 0.33 and 0.36 ± 0.24 pg/mL, respectively) concentrations were observed. In a cost-efficient fashion, the N-EBC method produced larger sample volumes, both pre- and post-exercise, facilitating more biomarker tests to be performed.

## 1. Introduction

Accurate and reliable biosample collection is essential for quantifying resultant biomarker assays. While recent innovations have overcome many of the barriers to the collection of biosamples (i.e., blood/plasma, saliva, biopsies, etc.), no single approach solves all the most common concerns. Among the critical barriers to collecting viable biosamples are the concerns related to obtaining adequate volume, sample viability, expense related to experimentation, and the invasive nature of the collection process. Based on these collective concerns, new research efforts aim to find alternative methods for collecting biological samples in a reliable, non-invasive, cost-effective fashion that is also applicable to clinical, laboratory, and field research settings. Notably, acute woodsmoke exposure in wildland firefighting has increased in capacity as of late, although the quantification of biomarker response that occurs during chronic exposures is less documented [1]. With respect to wildland fire settings, understanding stress at the air-lung interface is a critical next step in quantifying the physiological and biochemical consequences of acute smoke inhalation. Accordingly, there has been renewed interest in collecting exhaled breath condensate (EBC) as a medium for subsequent biomarker assay in recent years.

EBC collection is a non-invasive technique for obtaining large amounts of biosamples from the lower respiratory tract. EBC is obtained as breath is exhaled from the lungs into a cooled collecting device, condensing the vapor emerging with the breath, and commonly collected for 10 min time periods [2]. All nonvolatile compounds found in EBC originate in the airway lining fluid or are reaction products of volatiles. Fundamental to the critical concerns in biosample collection, the non-invasive procedure for EBC collection does not influence airway function or perturb the biochemical processes examined within resulting samples (e.g., inflammation). Because of the rapid, non-invasive nature of EBC collection, the technique is amenable to exercise and work physiology applications [3,4].

Among the primary reasons EBC is potentially useful to exercise and work physiology, the sample is directly influenced by the interface between the body and the environment, as experienced in the lungs. Accordingly, assays from EBC samples are promising for learning more about the pathobiology of various diseases and creating biochemical fingerprints of lung conditions in the future [3,5]. In addition, many inflammatory responses to environmental stressors (i.e., the introduction of airborne pathogens, inhalation of pollutants, etc.) are instigated by resident immune cells found in the capillary beds of the pulmonary system [6,7]. Based on this growing interest in EBC as a biological medium, commercial devices have proved successful in standardizing the EBC collection process [3]. Indeed, EBC has been measured for a number of years, with multiple commercially available devices established. Essential to this approach is the assurance that EBC samples do not contain saliva contamination (as evidenced by the presence of salivary amylase in levels found in human saliva), pH testing, and when sample volumes permit, the addition of a wide range of biomarkers. However, the use of EBC has remained limited in many experimental studies due to the cost of single-use devices and the limited sample volumes commonly collected.

Based on this rationale, the current study was undertaken as a proof-of-concept to develop a novel technique for collecting EBC in future investigations. The goals of the present study were to provide a method of novel EBC collection that achieved the following: (1) provide a more cost-effective method for obtaining viable EBC samples as compared to commercial devices; (2) to determine whether time-equivalent sample volumes obtained with the novel collection device were comparable to, or in excess of, those obtained with a commercially available device.

## 2. Materials and Methods

### 2.1. Participants

Before participant recruitment and testing, study approval was obtained from the University of Montana Institutional Review Board. Study participants (male: *n* = 10, female: *n* = 6) were recruited from the Missoula, Montana Community, and written informed consent was obtained before data collection. Each participant completed a personal information questionnaire. Study participants had no history of chronic lung disease or respiratory problems and were physically active. Before arrival at the lab, they confirmed they had not developed any respiratory infections or other health-related changes between recruitment and testing completion.

### 2.2. Study Design

**Figure 1:**
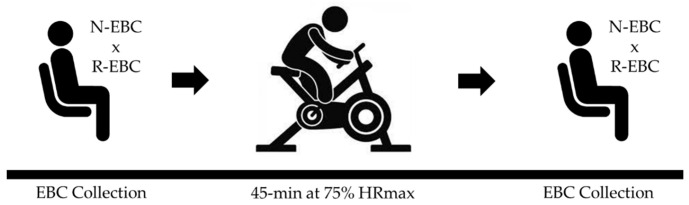
Experimental design. Exhaled breath condensate (EBC) collection (10 min) occurred immediately before and after the bout of exercise. A subset of participants (*n* = 5) completed both a 10 min N-EBC collection and a 10 min R-EBC collection in a randomized fashion both before and after the bout of exercise.

For the overall study design, participants (male: *n* = 10; female: *n* = 6; 26 ± 8 yrs.) completed a 10 min collection of breathing through a novel EBC collection device (N-EBC) while seated and wearing a nose clip. Sample size calculations (α= 0.05, 80% power) indicated *n* = 10 participants were needed based upon anticipated EBC sample volumes of 2.5 mL ± 1 mL [8]. In a subset of individuals (male: *n* = 3; female: *n* = 2), EBC was obtained using both the novel technique and a commercially available EBC collection device (R-EBC) in a randomized fashion (Figure 1). After the initial sample was obtained, participants completed a 45 min bout of cycling at 75% of their estimated HR max (roughly equivalent to 60% VO_2_ max) in between 10 min EBC collections in the study.

### 2.3. Commercial and Novel EBC Devices

**Figure 2:**
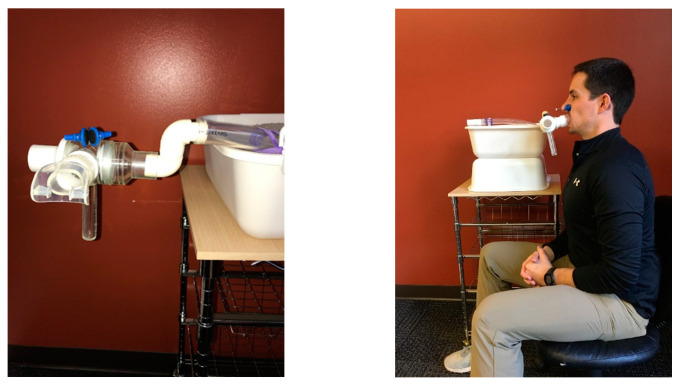
Device photos (front and side view). A Hans Rudolph mouthpiece equipped with a two-way nonrebreathing T-valve. The mouthpiece is connected to the 3/4″ Tygon Tubing via two 3/4″ PVC 90° Street Elbows, and a one-way valve is placed on the distal end of the tube.

For the N-EBC device, participants were asked to exhale “actively” into a 3/4″ diameter (1″ outer diameter, 3/4″ inner diameter, 1/8″ wall thickness) Tygon High-Purity tube while seated (US Plastics Corp., Lima, OH, USA). Except for the proximal and distal ends (5′ length), the EBC collection tube was submerged in an ice bath (0° Celsius) to cool the exhaled air and allow liquid collection. While wearing a nose clip, participants used a Hans Rudolph mouthpiece (Hans Rudolph, Inc., 8900 Series, Shawnee, KS, USA) equipped with a two-way nonrebreathing T-valve (Hans Rudolph, Inc., T-Shape Valve, Shawnee, KS, USA). The mouthpiece was connected to the EBC collection tube via two 3′4″ PVC 90° Street Elbows, and a one-way valve was placed on the distal end of the tube (Figure 2).

EBC biosample collection consisted of removing the Hans Rudolph mouthpiece, securing a 50 mL Falcon tube on the tube’s distal end, grasping the tubing’s proximal end, and spinning the tubing in a vertical plane. After circling the tubing apparatus at a speed of 60 rpm for one minute, the Falcon tube was oriented downward so that the EBC sample remained in the conical tip of the tube. Using a pipette, 0.7 mL samples of EBC were placed into 1.5 mL microcentrifuge tubes and stored until subsequent assay.

With the R-EBC device, participants repeated the 10 min seated active breathing protocol but instead exhaled into the RTube^TM^ breath condensate collection device (Respiratory Research, Inc., Austin, TX, USA). The device consists of a mouthpiece connected by a one-way valve into a collection tube. Per manufacturer instructions, the collection tube was surrounded by an aluminum sleeve pre-cooled in a −80° Celsius freezer. Using the plunger provided by the manufacturer, 0.7 mL samples of EBC were obtained by pipette, transferred into 1.5 mL microcentrifuge tubes, and stored until subsequent assay.

### 2.4. EBC Sample Storage, Handling, and Assay

EBC samples from both devices were placed on ice in a light-protected cooler (−20 °C). Within 3 min, samples were stored at −80 °C until subsequent assay, except for one microcentrifuge tube containing 1 mL of a subject’s sample used in pH testing. The pH testing (ThermoScientific Orion Star A111, Waltham, MA, USA) was conducted directly after sample collection per the manufacturer’s instructions to avoid alterations that may occur when samples are left open to room air (i.e., increases in pH due to CO_2_ and other dissolved gases lost into the atmosphere).

Salivary α-amylase was assayed using a kinetic enzyme assay kit (Salimetrics, LLC, State College, PA, USA). Similarly, oxidative stress biomarkers (8-isoprostane and Myeloperoxidase) were assayed using enzyme-linked immunoabsorbent assay (ELISA) kits (Cayman, Ann Arbor, MI, USA). Finally, a pro-inflammatory biomarker (Pentraxin-3) was assayed using an ELISA kit (R&D Systems, Inc., Minneapolis, MN, USA). All kits were performed in accordance with the manufacturer’s instructions.

### 2.5. Data Analysis and Statistical Testing

Data are reported as mean ± standard deviation for pre- and post-exercise time points. The primary objective of our statistical analyses was to evaluate if there was a relative change in each biomarker concentration following the exercise. Paired sample *t*-tests were performed to observe differences between test points, and variance comparisons were analyzed between device values. Results were classified as statistically significant when the *p*-value was 0.05 or less based upon *a priori* criteria. All analyses were performed using SAS Studio (SAS Institute Inc., Cary, NC, USA).

## 3. Results

Sixteen active individuals (male: *n* = 10; female: *n* = 6) participated in the study. Participant characteristics are presented in Table 1. N-EBC volume pre- and post-exercise (2.3 ± 0.8 and 2.6 ± 0.9 mL, respectively) and pH (7.4 ± 0.5 and 7.4 ± 0.5, respectively) were not significantly different. Furthermore, no gender differences were observed for N-EBC comparisons. When normalized for participant body height, device comparisons indicated N-EBC volumes were larger than corresponding samples R-EBC at pre-exercise (+12%) and post-exercise (+48%) (Table 2). Pre-exercise pH was significantly different between the N-EBC and R-EBC trials (7.4 ± 0.5 and 6.3 ± 0.2, respectively; *p* < 0.01), suggesting untoward chemical interactions with the R-EBC device (Table 2). N-EBC Salivary α-Amylase values were below the normal range for saliva samples and comparable to R-EBC (0.08 ± 0.56 and 0.21 ± 0.35 U/mL, respectively; *p* = 0.61).

Following the bout of moderate-intensity exercise, no changes in pre-and post-trial values of Pentraxin 3 (0.25 ± 0.04 and 0.26 ± 0.06 pg/mL, respectively; *p* = 0.44) and 8-Isoprostrane (0.43 ± 0.33 and 0.36 ± 0.24 pg/mL, respectively; *p* = 0.34) concentrations were observed (Table 3). Myeloperoxidase was only detectable in 7 of 30 samples (0.19 (range: 0.06–0.51) ng/mL), precluding further analysis.

## 4. Discussion

The current investigation compared a novel EBC collection device to a commercially available apparatus. The impetus for this investigation relates to our ongoing research into the potential for acute physiological and biochemical detriment following wood smoke exposure. In this regard, the EBC biosamples are reflective of the air–lung interface and may hold new insights into the stresses associated with smoke inhalation. Accordingly, EBC biosample volume and stability are central to the eventual understanding of acute smoke inhalation. Our findings indicate that both the N-EBC and the R-EBC collected with the RTube^TM^ apparatus were adequate for collecting EBC biosamples before and after a bout of moderate-intensity cycle ergometer exercise. In this regard, one of our study goals was to determine whether the N-EBC could provide equivalent EBC samples than RTube^TM^ but at a lower cost. In this investigation, our samples were collected for a total of USD 200, a value that would have cost more than USD 4000 for a comparable collection using the RTube^TM^ device. The other aim of this investigation was to determine whether the N-EBC would provide a larger volume biosample in a time-equivalent fashion. Findings indicate that when normalized for participant height (a critical anatomical predictor of pulmonary volumes and subsequent ventilatory rates at rest), N-EBC was +12% pre-exercise and +48% post-exercise compared to R-EBC. The larger post-exercise volume was likely due to elevations in post-exercise ventilatory rates (reflecting increases in both respiratory rate and tidal volumes) [9]. In addition, the novel EBC collection device has a significantly larger surface area as compared to the commercially available device, potentially increasing the EBC capture capacity. Accordingly, the elevated ventilatory rates and device surface areas combined to amplify between-device sample volumes in the post-exercise collection period. Interestingly, and given that EBC is almost invariably collected in 10 min time windows, the increased minute ventilation observed currently is likely resolved within 10 min of the cessation of moderate exercise [10]. Based on this understanding, it is serendipitous that the current methodology may have optimized the collection of larger sample volumes in the post-exercise time point.

Among the most critical factors related to EBC quality control is to collect a voluminous sample without salivary contamination. To achieve this end within a work physiology laboratory setting, we used a commonly available Hans Rudolph mouthpiece (including a saliva trap) attached to a PVC junction that facilitated the collection of exhaled water vapor but prevented the passage of saliva into the EBC collection tube. To confirm the absence of salivary contamination, EBC biosamples were assayed for the presence of salivary amylase. As with prior EBC investigations [4,11], our data indicated all samples contained amylase concentrations below the physiologic level of human saliva, which are typically lowest in pre-stress situations and exhibit peak values immediately post-stressor, a response profile that partially resembles salivary cortisol [12].

In the current study, levels of both salivary alpha-amylase and 8-isoprostane exhibited relatively large signal:noise ratios. This anticipated finding reflects the relatively low-stress nature of this exercise-based study. Moreover, in both EBC and saliva, concentrations of salivary amylase can differ dramatically within an order of magnitude, relative to the given biological medium. When evaluating the body of literature on saliva samples, those error values are widely variable in both physical and psychological (non-physical) trials. In non-exercise situations, Gordis et al. (2006) found salivary alpha-amylase values were 85.49 ± 64.31 U/mL and 114.39 ± 83.07 U/mL, before and after a psychological stress stimulus [12]. Similarly, following a 10 km running distance, Deneen and Jones (2017) found salivary alpha-amylase in male runners increased from approximately 90 ± 20 U/mL to 160 ± 50 U/mL [13]. In contrast, when comparing between EBC or saliva, salivary amylase concentrations differed by several orders of magnitude. Indeed, salivary amylase concentrations from our EBC samples were well below the levels associated with human saliva.

Similar to salivary amylase outcomes, the large signal:noise ratios for 8-isoprostanes reflect the low-stress nature of the exercise used in this investigation. In this instance, 8-isoprostanes were measured as a representative marker for post-exercise oxidative damage. Lipid markers of oxidative damage are typically proportional to the exercise intensity [14]. Thus, low levels of 8-isoprostanes were anticipated, likely elevating the signal:noise ratio for that metric. Moreover, for given sub-maximal exercise intensity, markers of lipid oxidative damage can be quite variable, a fact that appears to be true in both human blood and EBC samples [15,16].

Another impetus for the current investigation of our novel device was to demonstrate whether EBC biosamples could be collected in volumes that permitted the accurate determination of pH, in addition to a panel of stress and inflammation biomarkers. To prevent the potential for cross-contamination from the pH probe, cleaned between each sample per manufacturer instructions, it is ideal for the pH sample to be determined from a dedicated aliquot. In this regard, pH values collected from N-EBC samples were similar to the physiologic ranges from prior investigations [17,18,19,20], although exhibiting minimal differences in the post-exercise values. In contrast, pH values from R-EBC samples were significantly lower than typical physiological values and those obtained by the N-EBC. While it is possible that limiting sample volumes (i.e., lack of volume) could account for artifactual pH values, this was not likely the case in the current investigation where we examined pH using a 1 mL EBC sample volume for both devices. Accordingly, there is a rationale to suspect that R-EBC collected in contact with the aluminum sheathing (as necessitated by manufacturer protocols) of the R-tube may have resulted in artifactually low values. The device manufacturer’s guidelines recommend storing aluminum sleeves in a sealed bag to avoid moisture accumulation on the inside of the sleeve; however, the total absence of frost in the sleeve is not always attainable and could explain discrepant findings in pH between N-EBC and R-EBC samples. In contrast, N-EBC samples were collected in plasticizer-free tubing, perhaps explaining why pre- and post-exercise pH values taken from our novel device are similar to those observed in prior investigations [17,18,19,20].

Whether the current pH differences between N-EBC and R-EBC samples extend to bioassays is currently unknown. Nonetheless, it is understood from prior investigations that the accurate pH values from EBC samples can hold significant clinical and physiological importance beyond exercise applications. In support, previous investigations provide validated techniques and demonstrate physiologically relevant ranges (e.g., for healthy and diseased participants) for determining the pH from EBC [20]. Indeed, this method of EBC measurement is the most reproducible method for both healthy and asthmatic subjects [17,18]. In prior investigations, the mean pH of healthy subjects is 7.7, with a typical range of 7.4–8.8 [17]. Within post-exercise scenarios, alterations in airway pH reflect respiratory compensation to the elevated metabolic rate related to cycle ergometry.

In contrast, alterations in EBC pH from clinical (non-exercise) scenarios could reflect metabolic dysregulation and pathological processes unregulated underlying inflammatory diseases [21]. Moreover, in highly asthmatic individuals, a decrease in the pH of airways can cause bronchoconstriction and impairs multiple aspects of airway function [22]. Collectively, the current findings, combined with the existing literature, raise new insights into the importance of collecting EBC samples in inert receptacles and using standardized techniques to assure that accurate pH values reflect the metabolic condition of the subject and not an experimental artifact.

A final consideration for the current investigation was to devise a novel EBC collection technique that provides adequate sample volumes for the subsequent examination of a robust biomarker panel. For instance, a prior investigation by our research group collected EBC before and after exercise using commercially available EBC collection apparatus [23]. In this prior study, participants were exposed to low, moderate, and high inhaled concentrations of woodsmoke (0 μg∙m^−3^, 250 μg∙m^−3^, and 500 μg∙m^−3^, respectively). Woodsmoke contains various air pollutants such as carbon monoxide, respirable particulate matter, and other chemical compounds impacting large populations in the western United States [24,25,26]. In this regard, the lungs are the physiologic system for the human–environment interface. Moreover, since resident immune cells within pulmonary capillary beds are sensitive to smoke and other pollutants [27,28], EBC collection may serve as an essential biological media for quantifying acute physiologic stress. Acknowledging this point, the prevalence of wildfires in the summer months increases the likelihood of exposure to ambient wood smoke from wildfires [24,25,26]. Furthermore, recreational or occupational activity, such as wildfire management activities by wildland firefighters, may result in increased minute ventilation that could potentially exacerbate the magnitude of woodsmoke exposure [29]. Indeed, woodsmoke exposure has been associated with lung function decline in occupational populations such as wildland firefighters [30,31]. However, a limited number of studies have directly evaluated the acute response apparent in exhaled breath condensate. Chronic woodsmoke exposure is linked to adverse health effects on cardiovascular control and oxidative stress, although acute smoke inhalation effects are not well defined [32]. Furthermore, to better understand this response profile, a cost-efficient and repeatable technique are advantageous to examine the multitude of complexities in field research dynamics.

Accordingly, EBC volumes were small in our prior investigation, preventing comprehensive examination of a full biomarker panel [23]. Based on this rationale, current sample volumes using our novel EBC collection device were adequate for the assay of five biomarkers and potentially more, as indicated in another experimental design. Indeed, based on many commercial assay kit sample volumes (60–300 μL, including replicates), sample volumes collected in the current investigation would have supplied at least seven, and as many as thirty-six, biomarkers, depending on the assay panels chosen. Findings in this investigation did not produce significant exercise-dependent changes for the selected variables of 8-Isoprostrane, Pentraxin 3, and Myeloperoxidase. However, our primary intent was to demonstrate proof-of-concept for successfully examining stress-related biomarkers from a larger volume of EBC that were not saliva contaminated. Indeed, statistically insignificant findings following a relatively short duration, a moderate-intensity bout of exercise was not necessarily expected [16,23,32]. Instead, more extreme forms of exercise and the co-introduction of inhaled woodsmoke or other environmental stressors would have likely resulted in pre-to-post-exercise elevations in all the biomarkers examined currently [33,34,35].

## 5. Conclusions

Findings from this investigation support our study aims to demonstrate the efficacy of a novel EBC collection device that provides adequate sample volumes using a cost-effective approach. In this regard, our novel EBC device costs 10-fold less than a competing commercially available collection apparatus. Moreover, we demonstrated that our novel EBC device was comparable to a commercially available device and produced larger sample volumes when normalized to participant height. In addition, the sample volumes collected in this investigation were adequate for the subsequent assay of numerous biomarkers. Based on prior work by our research group and others, obtaining robust sample volumes is essential for sample viability and the subsequent collection of EBC biomarkers that reflect challenges inherent to the study design. In this regard, we observed that the pH of EBC samples collected from plasticizer-free tubing via our novel technique was in line with prior investigations, while the pH from the commercially available device was not. Finally, while our moderate-intensity, limited duration exercise challenge did not result in pre- or post-trial differences for the variables examined, we demonstrated a proof-of-concept approach to collect a panel of relevant biomarkers from EBC biosamples. Moreover, these samples can be obtained from laboratory or field settings using a time-efficient non-invasive approach. Accordingly, these findings suggest that a follow-up investigation of EBC samples using this novel collection technique, combined with significant physiologic and environmental challenges (e.g., exercise and woodsmoke inhalation), is warranted.

## Figures and Tables

**Table 1:** Participant Characteristics.

Characteristic	*n* = 16	Males (*n* = 10)	Females (*n* = 6)
Age (y)	26 ± 8	26 ± 9	28 ± 7
Height (cm)	176 ± 10	182 ± 4	166 ± 7
Mean Exercise Trial Heart Rate (bpm)	157 ± 9	156 ± 9	158 ± 10
Mean Exercise Trial Watt Output (W)	163 ± 49	189 ± 25	121 ± 53

Data presented as mean ± SD.

**Table 2:** Exhaled Breath Condensate (EBC) Collection Characteristics.

Device	Volume (mL)		pH	
	Pre-Trial	Post-Trial	Pre-Trial	Post-Trial
N-EBC (*n* = 16)	2.3 ± 0.8	2.6 ± 0.9	7.4 ± 0.5	7.4 ± 0.5
R-EBC (*n* = 5)	2.1 ± 0.7	1.8 ± 0.4	6.3 ± 0.2 *	6.2 ± 0.2 *

Data presented as mean ± SD. * Indicates statistical significance (*p* < 0.01).

**Table 3:** Variables of Oxidative Stress.

Marker	N-EBC	R-EBC
Salivary α-amylase (U∙mL^−1^)		
Pre	0.06 ± 0.06	0.02 ± 0.03
Post	0.08 ± 0.56	0.21 ± 0.35
8-Isoprostane (pg∙mL^−1^)		
Pre	0.43 ± 0.33	2.34 ± 2.77
Post	0.36 ± 0.24	1.71 ± 1.78
Pentraxin-3 (pg∙mL^−1^)		
Pre	0.25 ± 0.04	0.37 ± 0.04
Post	0.26 ± 0.06	0.38 ± 0.05

Data presented as mean ± SD. N-EBC, Novel exhaled breath condensate collection device; R-EBC, RTubeTM device.

## Data Availability

The data presented in this study are available on request from the corresponding author.

## References

[1] Adetona O., Reinhardt T.E., Domitrovich J., Broyles G., Adetona A., Kleinman M.T., Ottmar R.D., Naeher L. (2016). Review of the health effects of wildland fire smoke on wildland firefighters and the public. Inhal. Toxicol..

[2] ATS Workshop (2006). ATS Workshop Proceedings: Exhaled nitric oxide and nitric oxide oxidative metabolism in exhaled breath condensate: Executive summary. Am. J. Respir. Crit. Care Med..

[3] Grob N.M., Aytekin M., Dweik R.A. (2008). Biomarkers in exhaled breath condensate: A review of collection, processing and analysis. J. Breath Res..

[4] Hunt J. (2002). Exhaled breath condensate: An evolving tool for noninvasive evaluation of lung disease. J. Allergy Clin. Immunol..

[5] Carraro S., Rezzi S., Reniero F., Héberger K., Giordano G., Zanconato S., Guillou C., Baraldi E. (2007). Metabolomics Applied to Exhaled Breath Condensate in Childhood Asthma. Am. J. Respir. Crit. Care Med..

[6] Montuschi P., Mondino C., Koch P., Barnes P.J., Ciabattoni G. (2006). Effects of a leukotriene receptor antagonist on exhaled leukotriene E4 and prostanoids in children with asthma. J. Allergy Clin. Immunol..

[7] Shahid S.K., A Kharitonov S., Wilson N.M., Bush A., Barnes P.J. (2005). Exhaled 8-isoprostane in childhood asthma. Respir. Res..

[8] Soyer O.U., Dizdar E.A., Keskin O., Lilly C., Kalayci O. (2006). Comparison of two methods for exhaled breath condensate collection. Allergy.

[9] Joye H. (1968). Comparison of the rapid and slow components of the curve of oxygen consumption, of carbonic anhydride elimination and the curve of ventilation during the recovery period. Int. Z. Angew. Physiol..

[10] Williams R.E., Horvath S.M. (1995). Recovery from dynamic exercise. Am. J. Physiol..

[11] Zweifel M., Rechsteiner T., Hofer M., Boehler A. (2013). Detection of pulmonary amylase activity in exhaled breath condensate. J. Breath Res..

[12] Gordis E.B., A Granger D., Susman E.J., Trickett P.K. (2006). Asymmetry between salivary cortisol and α-amylase reactivity to stress: Relation to aggressive behavior in adolescents. Psychoneuroendocrinology.

[13] Deneen W.P., Jones A.B. (2017). Cortisol and Alpha-amylase changes during an Ultra-Running Event. Int. J. Exerc. Sci..

[14] Quindry J.C., Stone W.L., King J., Broeder C.E. (2003). The Effects of Acute Exercise on Neutrophils and Plasma Oxidative Stress. Med. Sci. Sports Exerc..

[15] Bloomer R.J., Fisher-Wellman K.H., Ferebee D.E., Quindry J.C. (2009). Postprandial oxidative stress: Influence of sex and exercise training status. Med. Sci. Sports Exerc..

[16] Peters B., Ferebee D.E., Fisher-Wellman K.H., Quindry J.C., Schilling B.K. (2018). Experimental Woodsmoke Exposure during Exercise and Blood Oxidative Stress. J. Occup. Environ. Med..

[17] Accordino R., Visentin A., Bordin A., Ferrazzoni S., Marian E., Rizzato F., Canova C., Venturini R., Maestrelli P. (2008). Long-term repeatability of exhaled breath condensate pH in asthma. Respir. Med..

[18] Kullmann T., Barta I., Lazar Z., Szili B., Barat E., Valyon M., Kollai M., Horvath I. (2007). Exhaled breath condensate pH standardised for CO2 partial pressure. Eur. Respir. J..

[19] Tuesta M., Alvear M., Carbonell T., García C., Guzmán-Venegas R., Araneda O.F. (2016). Effect of exercise duration on pro-oxidants and pH in exhaled breath condensate in humans. J. Physiol. Biochem..

[20] Vaughan J., Ngamtrakulpanit L., Pajewski T.N., Turner R., Nguyen T.A., Smith A., Urban P., Hom S., Gaston B., Hunt J. (2003). Exhaled breath condensate pH is a robust and reproducible assay of airway acidity. Eur. Respir. J..

[21] Powers S., Howley E., Quindry J. (2021). Acid-Base Balance during Exercise. Exercise Physiology: Theory and Application to Fitness and Performance.

[22] Holma B., Hegg P.O. (1989). pH- and protein-dependent buffer capacity and viscosity of respiratory mucus. Their interrelationships and influence of health. Sci. Total Environ..

[23] Ferguson M.D., Semmens E.O., Dumke C., Quindry J.C., Ward T.J. (2016). Measured Pulmonary and Systemic Markers of Inflammation and Oxidative Stress Following Wildland Firefighter Simulations. J. Occup. Environ. Med..

[24] Booze T.F., Reinhardt T.E., Quiring S.J., Ottmar R.D. (2004). A Screening-Level Assessment of the Health Risks of Chronic Smoke Exposure for Wildland Firefighters. J. Occup. Environ. Hyg..

[25] Dunn K.H., Shulman S., Stock A.L., Naeher L.P. (2013). Personal Carbon Monoxide Exposures Among Firefighters at Prescribed Forest Burns in the Southeastern United States. Arch. Environ. Occup. Health.

[26] Naeher L.P., Brauer M., Lipsett M., Zelikoff J.T., Simpson C., Koenig J.Q., Smith K.R. (2007). Woodsmoke Health Effects: A Review. Inhal. Toxicol..

[27] Rogers L.K., Cismowski M.J. (2018). Oxidative stress in the lung—The essential paradox. Curr. Opin. Toxicol..

[28] Rothman N., Ford D.P., Baser M.E., Hansen J.A., O’Toole T., Tockman M.S., Strickland P.T. (1991). Pulmonary function and respiratory symptoms in wildland firefighters. J. Occup. Med..

[29] Adetona O., Dunn K., Hall D.B., Achtemeier G., Stock A., Naeher L.P. (2011). Personal PM2.5Exposure Among Wildland Firefighters Working at Prescribed Forest Burns in Southeastern United States. J. Occup. Environ. Hyg..

[30] Adetona O., Hall D.B., Naeher L.P. (2011). Lung function changes in wildland firefighters working at prescribed burns. Inhal. Toxicol..

[31] Slaughter J., Koenig J.Q., Reinhardt T.E. (2004). Association Between Lung Function and Exposure to Smoke Among Firefighters at Prescribed Burns. J. Occup. Environ. Hyg..

[32] Williamson-Reisdorph C.M., Tiemessen K.G., Christison K., Gurney S., Richmond D., Wood K., Quindry T.S., Dumke C.L., Quindry J.C. (2021). Cardiovascular and Blood Oxidative Stress Responses to Exercise and Acute Woodsmoke Exposure in Recreationally Active Individuals. Wilderness Environ. Med..

[33] Barregard L., Sällsten G., Gustafson P., Andersson L., Johansson L., Basu S., Stigendal L. (2006). Experimental Exposure to Wood-Smoke Particles in Healthy Humans: Effects on Markers of Inflammation, Coagulation, and Lipid Peroxidation. Inhal. Toxicol..

[34] Chen H., Samet J.M., Bromberg P.A., Tong H. (2021). Cardiovascular health impacts of wildfire smoke exposure. Part. Fibre Toxicol..

[35] Schwartz C., Bølling A.K., Carlsten C. (2020). Controlled human exposures to wood smoke: A synthesis of the evidence. Part. Fibre Toxicol..

